# Vaginal metastasis of a Ewing sarcoma five years after resection of the primary tumor

**DOI:** 10.1186/2045-3329-1-9

**Published:** 2011-08-01

**Authors:** Noemie Vanel, Victoire Vierling, Jennifer Kreshak, Marco Gambarotti, Stefania Cocchi, Cristina Tranfaglia, Daniel Vanel

**Affiliations:** 1 Departement of Pathology, the Rizzoli Institute, Via del Barbiano 1/10, 40106, Bologna, Italy; 2 Departement of Nuclear Medicine, San Orsola Hospital, Via Giuseppe Massarenti, 940138 Bologna, Italy

**Keywords:** Ewing sarcoma, vaginal metastasis

## Abstract

A 35-year-old female presented with pain and swelling of the distal left radius. A diagnosis of Ewing sarcoma was made and she underwent neoadjuvant chemotherapy and surgery. Macroscopic viable areas remained on the map of the surgical specimen; as such, she was classified as a poor responder and received high dose adjuvant chemotherapy. She remained disease-free for five years, until age 40. A vaginal polyp was then detected during a routine gynaecologic examination. It was removed and histopathology revealed metastatic Ewing sarcoma.

To our knowledge, this is the first reported case of a vaginal metastasis of Ewing sarcoma.

## Introduction

Ewing sarcoma (ES) is a small blue round cell tumor belonging to the Ewing Family Tumour (EFT) together with Primary Neuroectodermal Tumour (PNET) and ASKIN tumor (of the thoracic wall). Eight hundred and ninety six cases have been reported in our institute since 1982. Ewing sarcoma has a distinct predilection for males and occurs in the first two decades of life in more than 75 percent of cases [1,2].

Metastases are frequent [3] and are mostly pulmonary and osseous, but can be found in various other locations.

We present here the first description of a vaginal metastasis of Ewing sarcoma.

## Case Report

**Figure 1:**
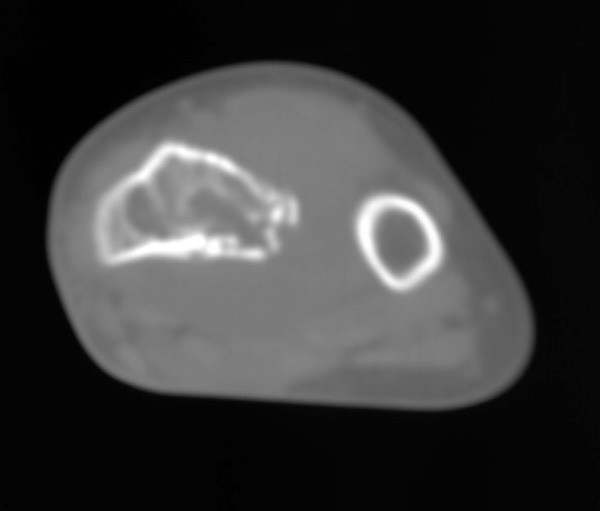
**Initial evaluation**. CT: lytic lesion with partial cortical destruction.

**Figure 2:**
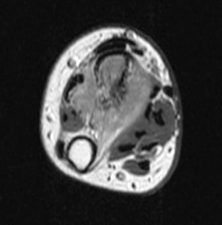
**Axial T1W MR image after contrast medium injection: the soft tissue extension is well studied**.

**Figure 3:**
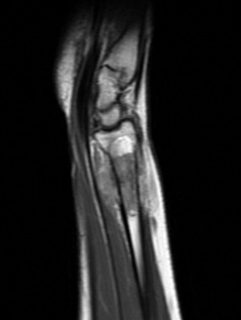
**Sagital T1W MR image, after contrast medium injection**. Medullary and soft tissue extensions are well evaluated.

A 35 year-old woman with no significant medical history presented with pain and swelling of the left wrist over the past year. A radiograph and computed tomography scan revealed a lytic lesion of the distal left radius (Figure 1), with soft tissue extension on MR examination (Figure 2, 3).

A biopsy was performed and histological examination revealed a typical Ewing sarcoma. The diagnosis was confirmed by FISH analysis, which demonstrated the translocation t (11, 22).

Staging revealed a solitary non-specific pulmonary nodule of the inferior right lobe that did not change with time and was not considered metastatic.

The patient underwent neoadjuvant chemotherapy and resection and allograft of the distal radius. She was considered as poor responder as macroscopic areas remained on the surgical specimen, but all margins were free of disease. High dose chemotherapy was then performed.

After completion of her treatment, it was followed up as per protocol and remained disease-free.

**Figure 4:**
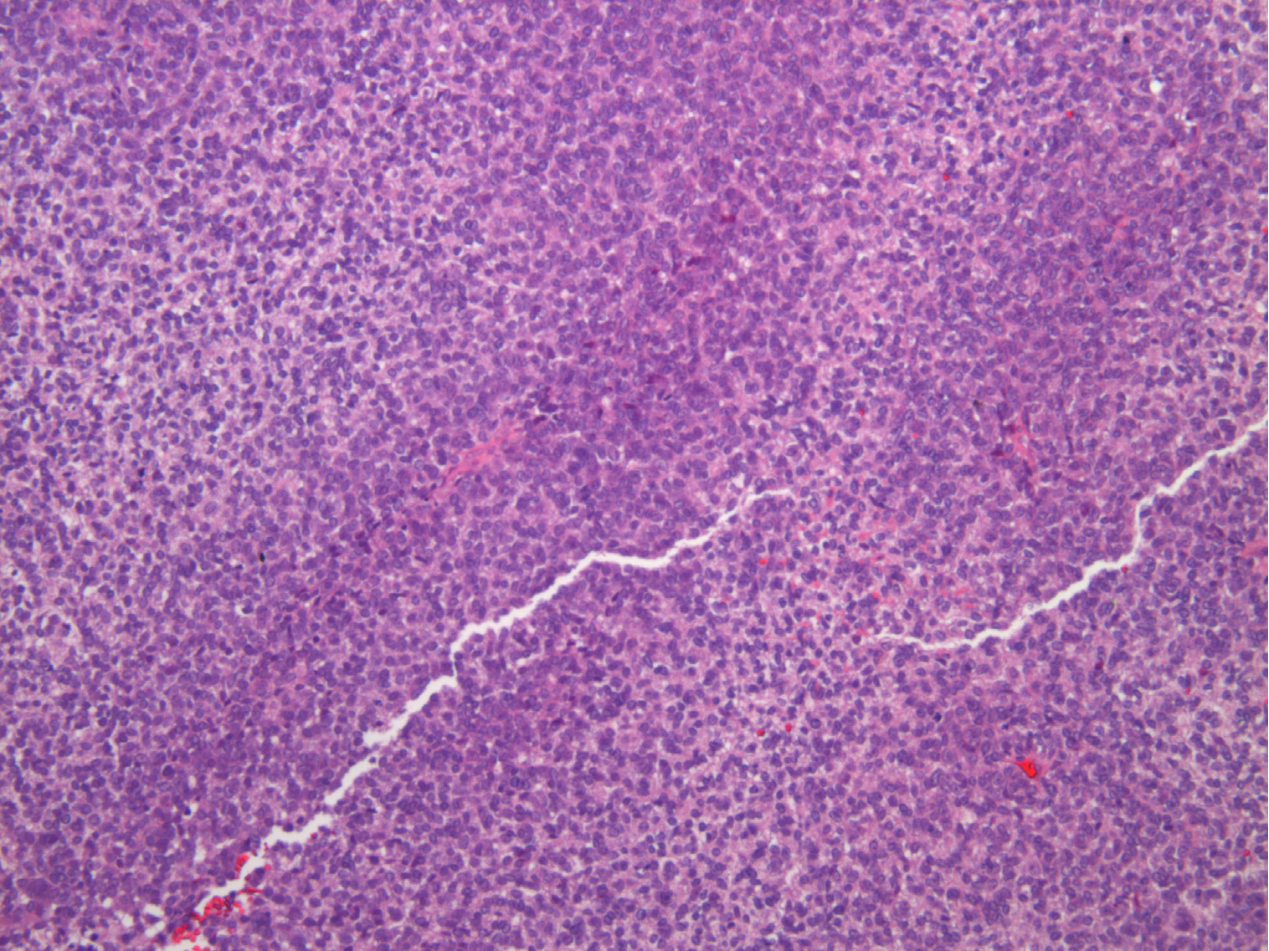
**The metastasis is made of homogeneous small round cells**.

**Figure 5:**
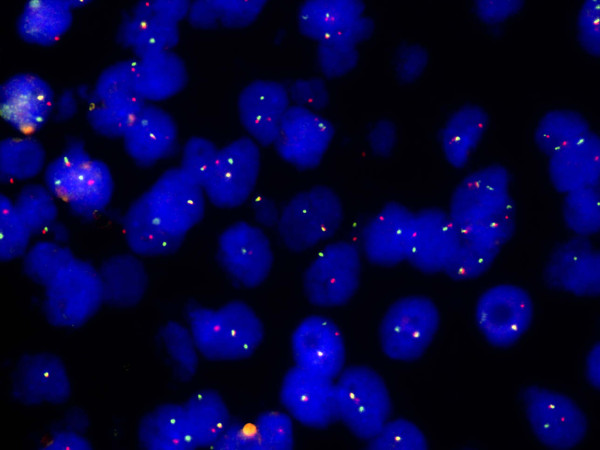
**Interphase FISH with the EWSR1 (22q12) break-apart probe**. Within a single nucleus fused red/green signals mark one intact 22q12 region, whereas split red/green signals indicate the presence of an EWSR1 gene rearrangement.

Five years later, during a routine gynaecologic exam, a vaginal polyp was found and removed. Histology revealed a ES metastasis (Figure 4), as confirmed by the characteristic translocation t(11, 22).(Figure 5). The rest of the evaluation (CT and bone scintigraphy) was normal.

No treatment has been undertaken.

## Discussion

Ewing sarcoma (ES) represents approximately ten percent[1] of primary malignant bone tumours and one percent of soft tissue tumours. It tends to arise in the diaphysis or metaphyseal-diaphyseal portion of long bones, although any bone may be involved.

Frequently, the first symptoms are pain and swelling.

Twenty percent of ES at diagnosis have radiographic evidence of metastasis. Lungs and bone are the main metastatic locations.

Radiologically, an aggressive osteolytic lesion is commonly observed.

ES is characterized by a morphologically uniform round cell proliferation with round nuclei containing fine chromatin.

CD99 is expressed in nearly all ES and thereby is a highly sensitive immunohistochemical marker.

Several studies have confirmed a characteristic 11, 22 (q24, q12) chromosomal translocation in 85 percent of the cases [3]; the translocation t (21, 22) and three even rarer translocations (t (7, 22), t(2, 22) t(17, 22)) have also been found.

Necrosis has a strong prognostic value [4]. High dose chemotherapy is used in poor responders [5].

We found 17 cases of primary ES involving the vagina and/or vulva in the literature[6]. A few cases of primary neuro ectodermal tumors (PNET) in the pelvis have also been reported [7].

Unusual metastasic locations have been described, for example, the breast [8], myocardial muscle [9], paranasal sinuses [10], iris [11]), and pancreas [12]. That explains why, even if a second primary cannot be completely excluded in our case, the probability of a metastasis is much higher. To our knowledge, this is the first case ever reported of a vaginal Ewing metastasis.

## Conclusion

This case exemplifies the idea that every new lesion in a patient with Ewing sarcoma should be considered as a possible metastasis.

## Competing interests

The authors declare that they have no competing interests.

## Authors' contributions

NV wrote the article, VV checked the case, JK corrected the writing (English and scientific content) MG checked and selected the histology, SC checked and selected the FISH, CT checked the scientific content, DV proposed the subject and directed the article. All authors read and approved the final manuscript
